# A Rare Case of Primary Pulmonary Choriocarcinoma Metastasizing to the Kidney: Diagnostic and Therapeutic Complexities

**DOI:** 10.1002/cnr2.70578

**Published:** 2026-05-15

**Authors:** Zorán Belics, Antónia Fürich, Levente Bogyó, Hanna Tihanyi, Mária Madarász, Dorottya Rózsa, Gizella Molnár, Balázs Gérecz, Petronella Hupuczi

**Affiliations:** ^1^ Széchenyi István University Győr Hungary; ^2^ Maternity Private Department of Obstetrics and Gynecology Budapest Hungary; ^3^ Department of Thoracic Surgery National Oncology Institute Budapest Hungary; ^4^ Semmelweis University Budapest Hungary

**Keywords:** beta‐hCG, chemotherapy, gestational trophoblastic disease, immunotherapy, primary pulmonary choriocarcinoma, renal metastasis

## Abstract

**Background:**

Choriocarcinoma is a highly aggressive malignant tumor composed primarily of cytotrophoblast and syncytiotrophoblast cells without villi and is characterized by beta‐human chorionic (beta‐hCG) gonadotropin production. Although most cases are of gestational origin, rare non‐gestational choriocarcinomas may arise from pluripotent germ cells in extragenital locations, independently of pregnancy. Primary pulmonary choriocarcinoma is extremely rare, and kidney metastasis has only exceptionally been reported in the literature.

**Case:**

We present the case of a 43‐year‐old patient with this unusual presentation. The diagnostic process began with a suspected implantation of unknown location and culminated in the final histopathological diagnosis based on lobectomy and nephrectomy specimens, including molecular genetic identification of the pulmonary tumor. Following thoracic and renal surgery, the patient received adjuvant chemotherapy and immunotherapy with anti‐PD‐1 monoclonal antibodies.

**Conclusion:**

This case highlighted the diagnostic challenges posed by primary pulmonary choriocarcinoma with renal metastasis, an exceptionally rare clinical entity. Ongoing follow‐up includes regular imaging studies and beta‐hCG monitoring.

## 1 Introduction

Choriocarcinoma is a highly vascular and invasive tumor composed of anaplastic trophoblasts, specifically cytotrophoblasts and syncytiotrophoblasts without villi. The tumor can be classified as either gestational or non‐gestational based on its origin. Non‐gestational choriocarcinoma may arise from germ cells or develop in association with high‐grade somatic malignancies [1]. Both types produce beta‐human chorionic gonadotropin (beta‐hCG) hormone.

The incidence of choriocarcinoma varies geographically, occurring in 1/30000 births in Europe and 1/1000 births in Asia. Among gestational choriocarcinomas, 80% develop from molar pregnancies, 15% follow miscarriage or childbirth, and approximately 5% originate in the ovaries. Following a molar pregnancy, malignant transformation of residual trophoblast cells can occur after a variable latency period ranging from weeks to years. The tumor frequently metastasizes to highly vascularized organs, including the brain, lungs, and liver.

Non‐gestational choriocarcinoma, a pluripotent germ cell tumor, can affect both sexes. In women, it typically occurs post‐menopause, with the primary tumor developing in extragenital organs such as the bladder, lung, mediastinum, or liver. In men, it usually originates from testicular germ cells and commonly presents in extragenital locations [2].

Primary pulmonary choriocarcinoma is particularly rare, with fewer than 40 cases reported in the medical literature [3, 4, 5, 6, 7, 8]. This case report details the diagnostic process of an implantation of unknown location that led to the discovery of this rare diagnosis, managed through a multidisciplinary approach.

## 2 Case Report

### 2.1 Ethics Statement

**FIGURE 1:**
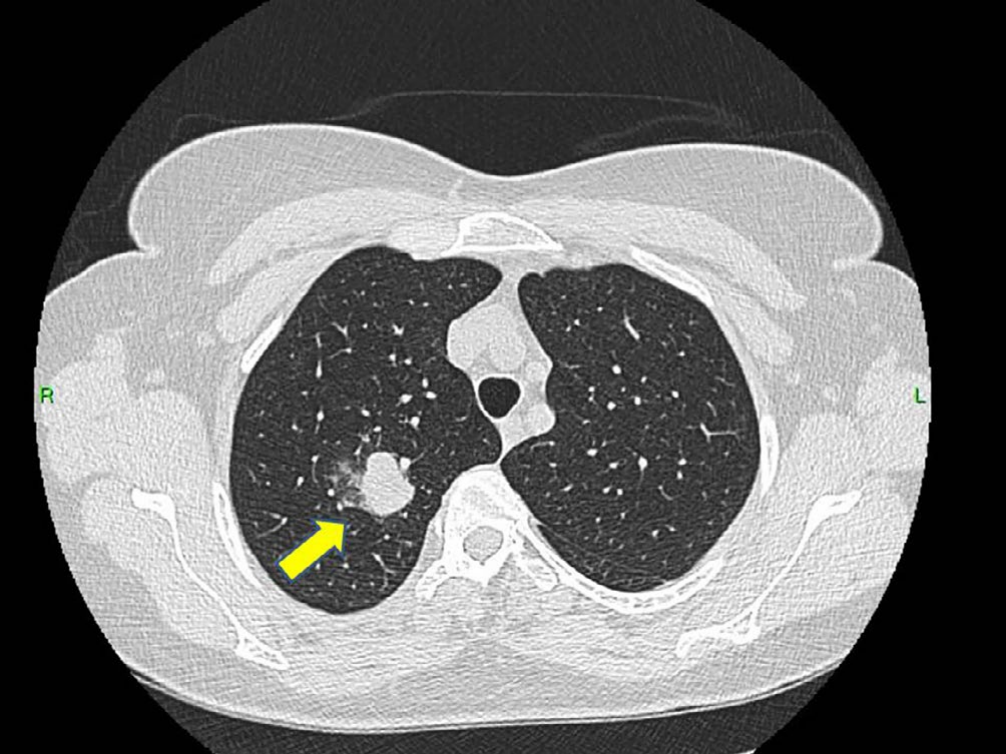
Chest computed tomography (CT) scan showing a solitary pulmonary nodule in the right upper lobe (yellow arrow), which was one of the first radiological findings raising suspicion of a thoracic malignant process.

**FIGURE 2:**
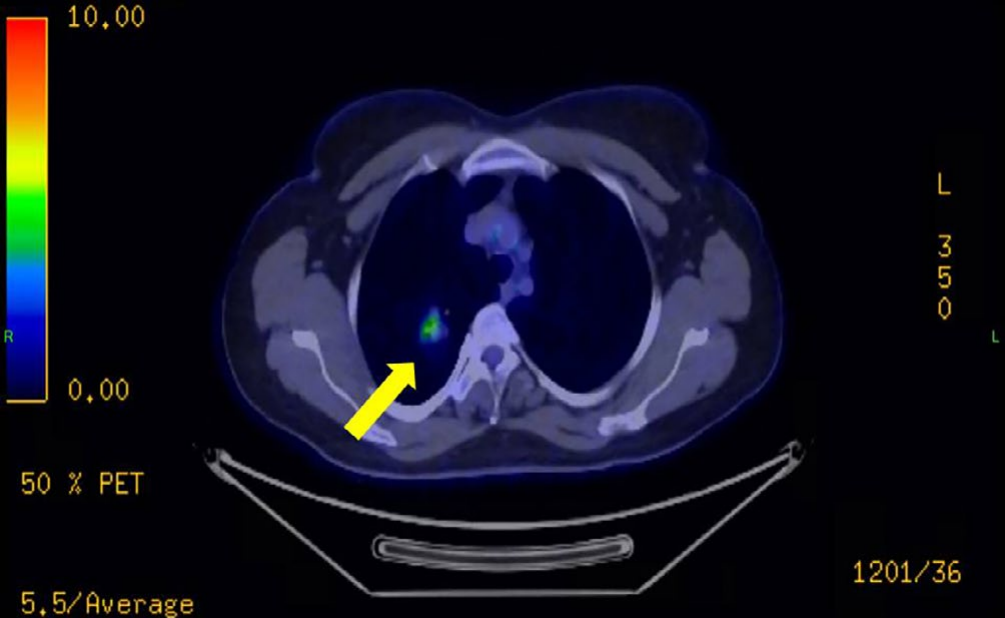
Fluorodeoxyglucose positron emission tomography‐computed tomography (FDG PET‐CT) scan demonstrating a metabolically active malignant lesion in the right upper lobe of the lung (yellow arrow), consistent with the primary pulmonary tumor.

**FIGURE 3:**
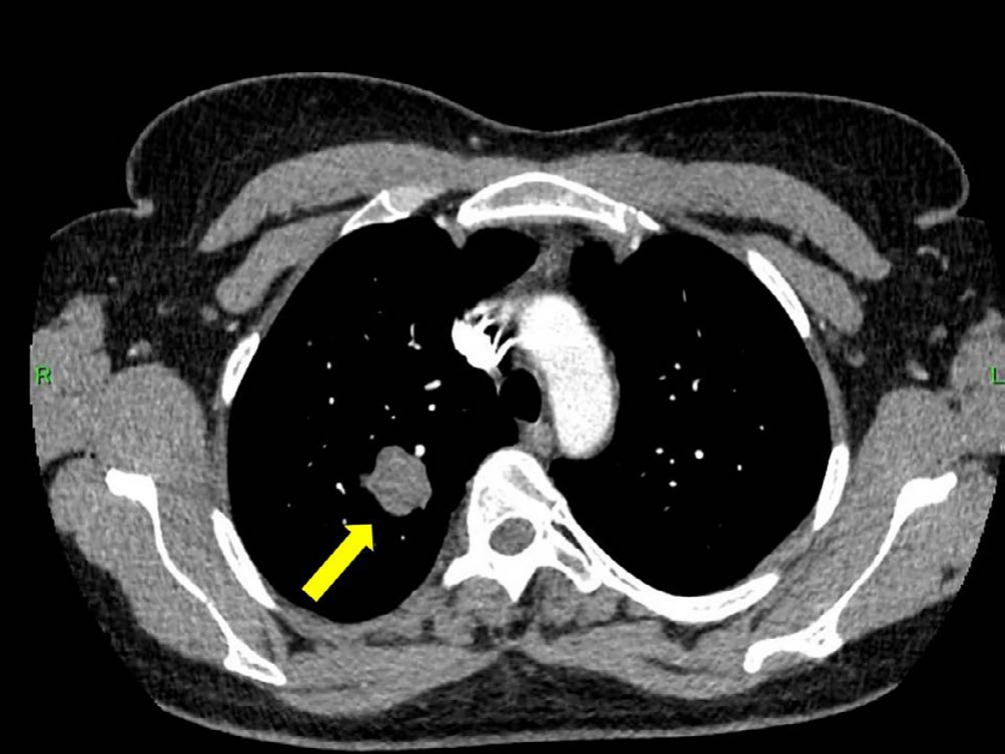
Chest CT angiography showing a malignant tumor in the right upper lobe of the lung (yellow arrow), further characterizing the thoracic lesion prior to surgical management.

**FIGURE 4:**
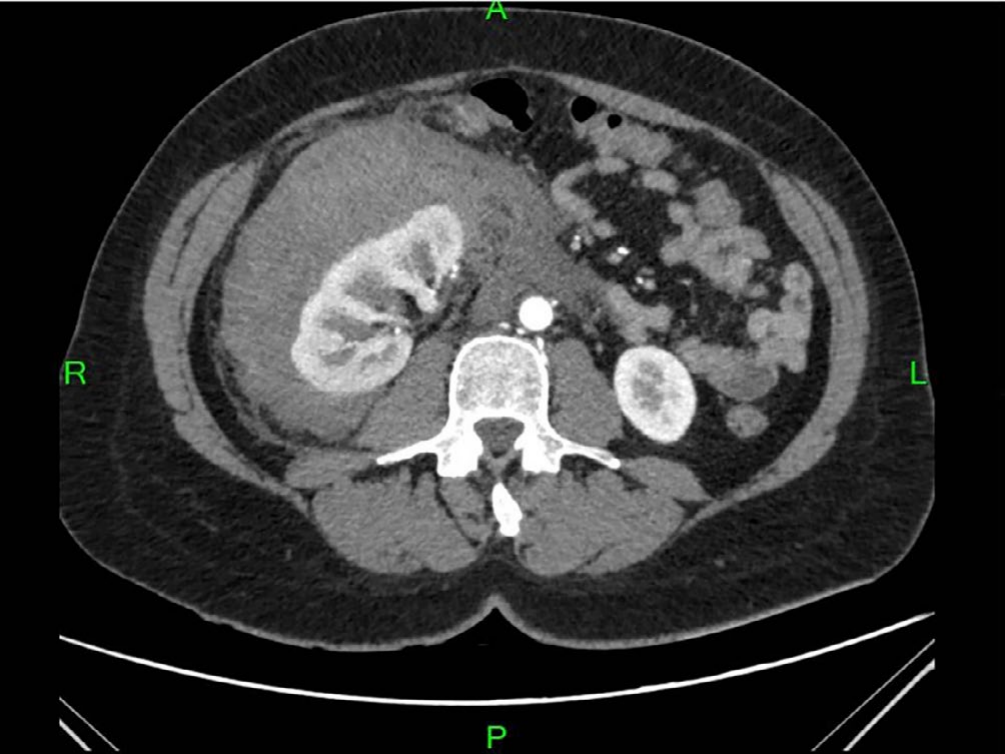
Contrast‐enhanced abdominal CT scan showing a hypodense parenchymal lesion in the right kidney with active contrast extravasation, associated with a large perirenal hematoma extending into the anterior and posterior pararenal spaces, consistent with hemorrhagic renal metastatic involvement.

**FIGURE 5:**
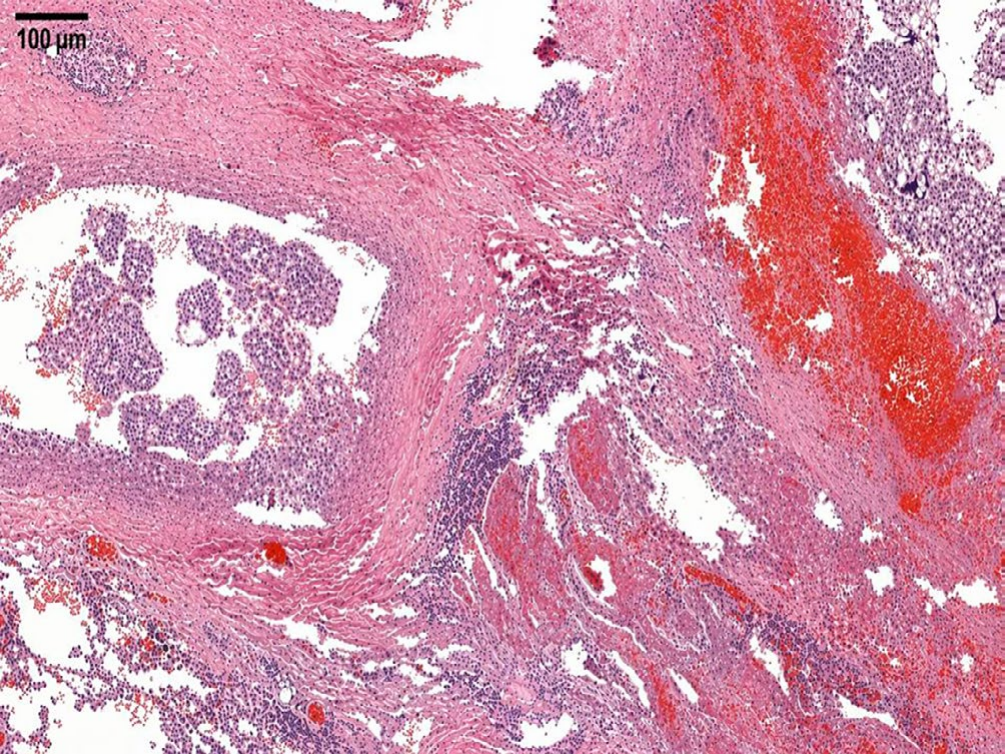
Histopathological examination of the resected lung tumor. Hematoxylin and eosin (H&E)‐stained sections show a hemorrhagic tumor composed of atypical multinucleated giant cells, syncytiotrophoblasts, and mononuclear intermediate trophoblasts with frequent mitotic figures (x56). Immunohistochemical staining for beta‐human chorionic gonadotropin (beta‐hCG) highlights the syncytiotrophoblastic component.

**FIGURE 6:**
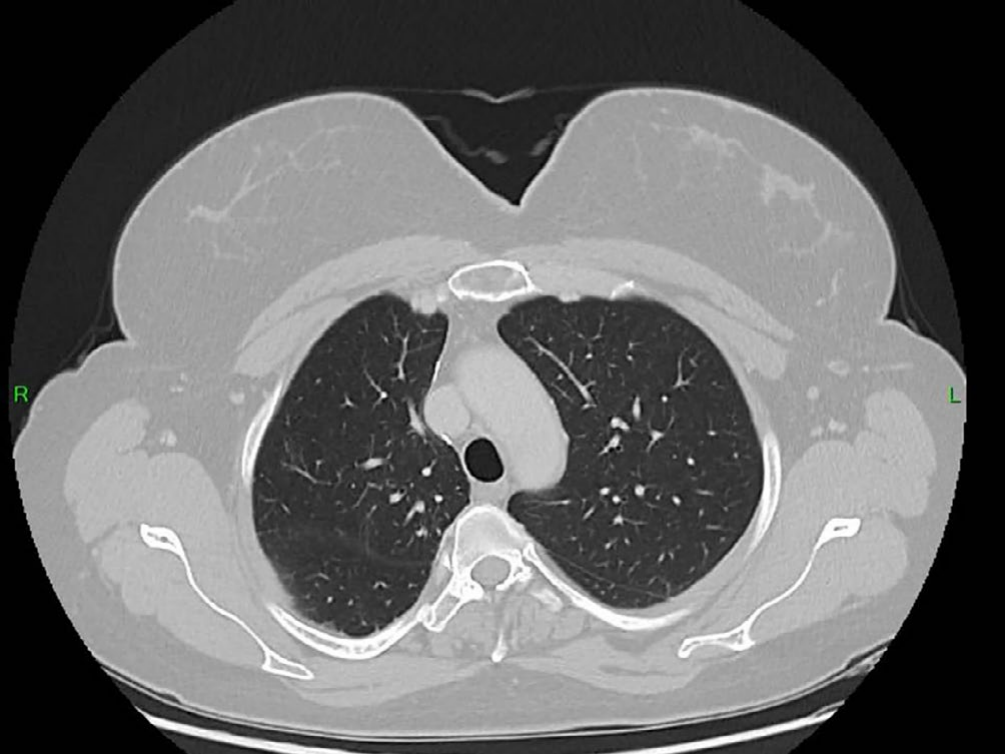
Follow‐up chest CT image obtained 18 months after video‐assisted thoracoscopic surgery (VATS) with right upper lobectomy and lymphadenectomy, and before initiation of pembrolizumab immunotherapy, showing no radiological evidence of recurrent thoracic disease.

**FIGURE 7:**
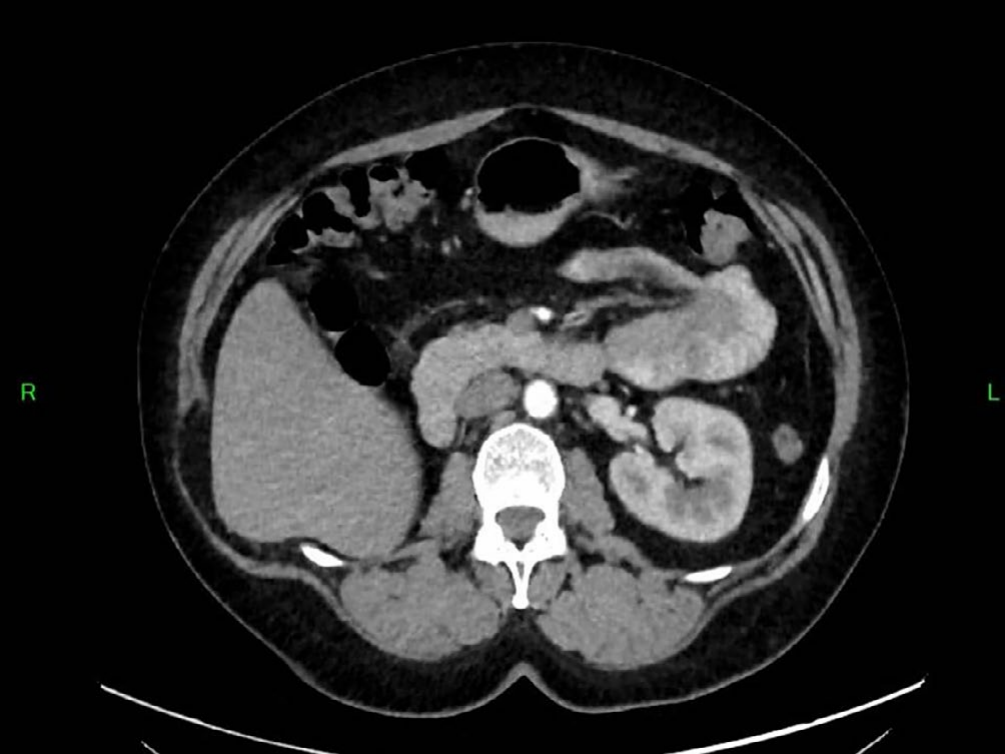
Follow‐up abdominal CT scan obtained 19 months after right nephrectomy, showing no abnormal findings and no radiological evidence of recurrent or residual abdominal disease.

We obtained written informed consent from the patient for publication of this case study and associated images (including Figures 1, 2, 3, 4, 5, 6, 7). The patient was informed that his identity would remain anonymous.

In March 2021, a 43‐year‐old woman presented to Maternity Private Department of Obstetrics and Gynecology, Budapest, Hungary with a 53‐day amenorrhea and positive pregnancy test, despite regular menstrual cycles. Her medical history was notable for one previous uncomplicated pregnancy resulting in delivery via cesarean section. Transvaginal ultrasound revealed a normal‐sized uterus with regular 10 mm endometrial thickness and bilaterally normal adnexa. Despite the missed period and positive pregnancy test, we could not confirm either intrauterine or extrauterine implantation, necessitating further investigation.

Initial laboratory testing revealed markedly elevated serum beta‐hCG levels of 3189 IU/L (normal value in non‐pregnant patients < 5 IU/L), which continued to rise to 3481 IU/L after 2 days. Diagnostic curettage was performed, and histopathological examination of the endometrial tissue showed proliferative endometrium without evidence of chorionic or decidual tissue, and no cellular atypia.

Prior to planned diagnostic laparoscopy, the patient developed new symptoms including hemoptysis and right lower back pain leading to the performance of a chest CT scan. This scan revealed a solitary nodule in the right upper lobe of the lung (Figure 1). Given the elevated levels of β‐HCG, choriocarcinoma was considered as a potential diagnosis (during this period, serum beta‐hCG levels continued to rise, reaching 4273 IU/L). However, due to the presence of hemoptysis, a biopsy could not be performed, and thoracic surgery was planned on a relatively urgent basis. For staging purposes, an urgent PET‐CT scan was conducted, which revealed a malignant tumor in the right upper lobe, as well as the CT angiography of the chest (Figures 2 and 3), but the PET‐CT scan did not show any pathological lesions outside the lung. The kidneys also appeared normal, although the diagnostic value of a PET‐CT scan is somewhat limited in evaluating this region. After the PET‐CT, the patient was scheduled for a right upper lobectomy. Nevertheless, a few days before the planned surgery, the patient experienced sudden abdominal pain and hematuria. An emergency abdominal angio‐CT confirmed bleeding from the right kidney. The bleeding from the renal metastasis was clinically significant. The acute abdominal CT showed a hypodense parenchymal lesion in the right kidney with active contrast extravasation, accompanied by a perirenal hematoma extending into the anterior and posterior pararenal spaces, causing a mass effect and elevating the liver (Figure 4). The kidney was malrotated due to compression, and the renal vessels were distorted. These findings indicate substantial hemorrhage, which justified the urgent nephrectomy, which was performed (histopathological examination confirming choriocarcinoma metastasis). Following this emergency procedure, and considering the hemoptysis, video‐assisted thoracoscopic surgery was undertaken to remove the right upper lobe along with regional lymphadenectomy also conducted as a second step.

The histological specimen from the lung was evaluated by the specialist in pulmonary pathology ‐Department of Surgical and Molecular Pathology at the Tumor Pathology Center, National Institute of Oncology, Budapest, Hungary (Figure 5). Post‐operatively, serum beta‐hCG levels decreased to 827 IU/L.

Molecular genetic testing was performed (DNA isolation method: Promega, Maxwell RSC DNA FFPE kit, DNA concentration, quality (ng/mL): (I) 27.9; (II) 39 ng/μL, Short tandem repeat (STR) assay Qiagen ESSPlex SEQS kit: Samples I and II are identical) to differentiate between choriocarcinoma of gestational and germ cell origin. The absence of identifiable paternal alleles in the tumor tissue suggested non‐gestational origin. The patient then received four cycles of combination chemotherapy with bleomycin, etoposide, and cisplatin, resulting in a reduction of serum beta‐hCG to 3 IU/L.

Follow‐up included regular CT examinations (Figures 6 and 7) together with serial laboratory assessment, particularly serum beta‐hCG monitoring. After 1 year, following a gradual increase in hormone levels to 64 IU/L, pembrolizumab immunotherapy was initiated. This intervention successfully reduced serum beta‐hCG to 30 IU/L. Following 11 cycles of immunotherapy, the patient maintains good general condition with stable low serum beta‐hCG levels (10–20 IU/L). Follow‐up bimanual gynecological examination and imaging studies 1 year post‐treatment completion showed no evidence of disease. The Table 1. provides a clear, chronological overview of the patient's beta‐hCG levels, imaging findings, and treatment modalities at each stage of patient diagnosis and management.

**TABLE 1:** The table provides a clear, chronological overview of the patient's beta‐hCG levels, imaging findings, and treatment modalities at each stage of patient diagnosis and management.

Timeline	Beta‐hCG level (IU/L)	Imaging findings	Treatment modalities	Remarks
Initial presentation	3189	Transvaginal ultrasound: Normal uterus, 10 mm endometrial thickness, normal adnexa	Ultrasound and Beta‐hCG follow‐up	No evidence of intrauterine or extrauterine pregnancy
Two days after	3481	No significant imaging changes	Diagnostic curettage	Rising beta‐hCG levels, no chorionic/decidual tissue on histopathology
Pre‐laparoscopy	4273	CT angiography/CT: Malignant tumor in right upper lobe, hemorrhagic cysts in kidney	None	New symptoms: hemoptysis, right lower back pain
Post‐nephrectomy	827	Histopathology: Choriocarcinoma metastasis in kidney	Right nephrectomy	Confirmed kidney metastasis
Post‐thoracic surgery	827	histopathological examination, molecular genetic testing: germ cell origin choriocarcinoma, the absence of identifiable paternal alleles in the tumor tissue suggested non‐gestational origin	VATS (right upper lobe resection, lymphadenectomy)	Confirmed lung primary tumor
Post‐chemotherapy	3	CT imaging: No evidence of disease, drug‐induced interstitial lesion	4 cycles of BEP chemotherapy (bleomycin, etoposide, cisplatin)	Rest period
1‐year follow‐up	64	CT imaging: No evidence of disease	Initiation of pembrolizumab immunotherapy	Gradual increase in beta‐hCG levels, prompting immunotherapy
Under immunotherapy	30	CT imaging: Stable, no disease progression	11 cycles of pembrolizumab	Beta‐hCG levels stabilized
Following 11 cycles of immunotherapy	14	CT imaging and bimanual gynecological exam: No evidence of disease		Patient in good general condition, stable low beta‐hCG levels

No adverse or unanticipated events occurred during the follow‐up period. At the latest follow‐up, the patient remained in good general condition with preserved quality of life and required no further active treatment.

## 3 Discussion

The combination of missed menstruation and positive pregnancy test typically suggests pregnancy, with transvaginal ultrasound serving as the primary tool for confirming implantation location. In cases where implantation cannot be visualized either intrauterine or extrauterine (pregnancy of unknown location), protocol dictates serial ultrasound examinations and hormone level monitoring. The diagnostic algorithm may include surgical procedures such as endometrial ablation and laparoscopy. In our case, histological examination of the endometrium excluded both intrauterine and classical ectopic implantation.

In case of elevated hCG, it is important to provide an analysis of rival diagnoses, including ectopic pregnancy and pregnancy of unknown location, gestational trophoblastic disease (GTD), and germ cell tumors. Ectopic pregnancy and pregnancy of unknown location, a common differential diagnosis in cases of elevated beta‐hCG levels without intrauterine pregnancy, were ruled out in our patient due to the absence of chorionic or decidual tissue on histopathological examination of the diagnostic curettage and the lack of adnexal abnormalities on transvaginal ultrasound imaging. Gestational trophoblastic disease, including molar pregnancy and gestational choriocarcinoma, was excluded based on the absence of paternal alleles in molecular genetic testing, confirming a non‐gestational origin [9, 10, 11]. Germ cell tumors, which can also present with elevated beta‐hCG levels and metastatic disease, were considered unlikely due to the absence of primary gonadal lesions and the distinct histopathological features of choriocarcinoma in the lung and kidney. By systematically addressing these differential diagnoses and providing a clear rationale for their exclusion, the diagnostic process is strengthened, and the validity of the final diagnosis is reinforced.

When these investigations prove inconclusive, clinicians should consider non‐gestational choriocarcinoma of ectopic origin [12, 13, 14, 15].

Planned laparoscopy was deferred due to the patient's unexpected symptoms suggesting extragenital origin, leading to the utilization of advanced imaging techniques including CT angiography and PET‐CT [16].

Non‐gestational choriocarcinomas typically carry a worse prognosis than their gestational counterparts, characterized by rapid progression and frequent presence of distant metastases at diagnosis [17]. In our patient, the presenting symptoms of hemoptysis and progressive lower back pain led to the discovery of concurrent lung and kidney involvement. While renal metastasis is uncommon even in gestational choriocarcinoma [18], renal metastasis from PPC appears to be exceptionally rare, with only very few reported cases.

Current evidence, including our experience, suggests that optimal treatment consists of surgical resection followed by adjuvant chemo‐ and immunotherapy [19]. Our patient received sequential chemotherapy protocols (BEP and EMA‐CO). As with other malignancies, early diagnosis significantly impacts treatment efficacy and survival rates [20]. In our case, the interval from initial presentation to diagnosis and treatment of extragenital choriocarcinoma was relatively brief.

Immunotherapy has emerged as an increasingly important treatment modality for malignant tumors. Checkpoint inhibitors, such as pembrolizumab, can effectively restore host immunity by targeting the interaction between programmed cell death protein 1 (PD‐1) on T‐cells and its ligand (PD‐L1) on tumor cells. The successful use of pembrolizumab in our patient demonstrates the potential value of immunotherapy in managing this rare malignancy [21].

While the patient is currently in a stable condition with low beta‐hCG levels (10–20 IU/L) and no evidence of disease on imaging, it is important to discuss predicted long‐term outcomes and compare them to potentially similar cases in the literature. Non‐gestational choriocarcinoma is associated with a high risk of relapse and poor long‐term survival due to its aggressive nature and frequent metastatic spread at diagnosis. The available studies suggest that the 5‐year survival rate for non‐gestational choriocarcinoma is approximately 30%–50%, significantly lower than that of gestational choriocarcinoma [22, 23]. Relapse rates are also high, with up to 40%–60% of patients experiencing recurrence, often within the first 2 years post‐treatment [24]. In our patient, the use of multimodal therapy, including surgery, chemotherapy, and immunotherapy, may improve long‐term outcomes compared to cases reported in the available literature. However, close monitoring of beta‐hCG levels and imaging studies is essential to detect early signs of relapse. Further studies are needed to establish standardized follow‐up protocols and evaluate the impact of immunotherapy on long‐term survival in non‐gestational choriocarcinoma.

Compared with the report by Abu Aljaaz et al. [25], our case illustrates a different and more gynecologically deceptive diagnostic pathway. Abu Aljaaz et al. described a woman initially misdiagnosed with lung adenocarcinoma, in whom FDG PET/CT revealed an aggressive metastatic pattern including renal involvement. In contrast, our patient first presented with amenorrhea, a positive pregnancy test, persistently elevated beta‐hCG levels, and no evidence of intrauterine or extrauterine pregnancy, thereby mimicking pregnancy of unknown location rather than primary thoracic malignancy. Our case is further distinguished by hemorrhagic renal metastasis requiring urgent nephrectomy and by molecular genetic testing demonstrating the absence of identifiable paternal alleles, which strongly supported a non‐gestational origin.

## 4 Conclusion

When elevated serum beta‐hCG levels are present without ultrasonographic evidence of intrauterine or extrauterine implantation, clinicians should consider non‐gestational choriocarcinoma with extragenital localization. While primary pulmonary choriocarcinoma remains rare, our case demonstrates that modern imaging techniques can facilitate diagnosis, and contemporary treatment approaches combining surgery with targeted immunotherapy can achieve favorable outcomes. This case contributes to the limited literature on this unusual presentation and highlights the importance of considering extragenital choriocarcinoma in the differential diagnosis of elevated beta‐hCG with pregnancy of unknown location.

The most significant finding of this case is that primary pulmonary choriocarcinoma presented as a pregnancy of unknown location, creating a substantial diagnostic pitfall. The case is further distinguished by the exceptional rarity of renal metastasis and by the use of molecular genetic testing to confirm non‐gestational origin through the absence of paternal alleles. The main challenges were the broad differential diagnosis of elevated beta‐hCG, the difficulty of identifying the primary site in an initially gynecologic clinical context, and the lack of standardized treatment recommendations for this rare tumor. Our experience suggests that timely multidisciplinary management, including surgery, platinum‐based chemotherapy, and immunotherapy, may achieve meaningful disease control, but close biochemical and radiologic follow‐up remains essential because of the high risk of relapse.

## Author Contributions

**Zorán Belics:** conceptualization, investigation, funding acquisition, writing – original draft, methodology, validation, visualization, writing – review and editing, software, formal analysis, project administration, data curation, supervision. **Antónia Fürich:** writing – review and editing. **Levente Bogyó:** writing – review and editing, investigation. **Hanna Tihanyi:** writing – review and editing, investigation. **Mária Madarász:** formal analysis. **Dorottya Rózsa:** formal analysis. **Gizella Molnár:** formal analysis. **Balázs Gérecz:** formal analysis. **Petronella Hupuczi:** formal analysis, writing – review and editing, investigation.

## Funding

The authors have nothing to report.

## Consent

Written informed consent was obtained from the patient for publication of the case details and accompanying images (including Figures 1, 2, 3, 4, 5, 6, 7). The patient was informed that his identity would remain anonymous and signed an informed consent form approved by the Institutional. A copy of the signed informed consent form is available from the corresponding author upon request.

## Conflicts of Interest

The authors declare no conflicts of interest.

## Data Availability

The data that support the findings of this study are available from the corresponding author upon reasonable request.
